# The Middlesex Hospital Appeal

**Published:** 1910-10-01

**Authors:** 


### The Middlesex Hospital Appeal.

The appeal in aid of the Middlesex Hospital, for the reduction of the debt of £20,000 resting on the institution, has met with prompt and signal success upon which everyone connected with the venture is to be heartily congratulated. In his circular letter to the Press, Prince Francis of Teck, the indefatigable Chairman of the Board of Governors, points out that there still remains the accumulated deficit between income and expenditure for three years to be wiped off before the Middlesex can be said to be secure upon a sound financial basis. A large general hospital like this needs a permanent and steady income if it is to progress along lines of modern institutional development. To subject it to the strain of periodical appeals for temporary help, in consequence of a fluctuant and uncertain income, is to rob it very largely of all incentive to expansion and improvement. The West End hospital, existing as it does in a comparatively rich quarter where its work is, as it were, continually under the eyes of its patrons, has a large circle of friends, and we are confident that they will not be behindhand in expressing their sympathy towards the contemplated endowment fund in as practical and effective a manner as they have done in the matter of erasing from the hospital's books that debt of £20,000.

The success of the recent appeal teaches two lessons which hospital authorities all over the country will do well to take to heart. One of these is the vitality of voluntary charity. Of late years a vast amount of nonsense has been written and said about the breakdown of the whole structure of voluntary philanthropy upon which this country has hitherto prided itself. Allegations have been made to the effect that the nation is growing apathetic, that it is no longer responsive to the argument of humanity, no longer capable of exercising a just and fair discretion in the distribution of its charity. If proofs were wanted of the falsity of such statements, the signal success of recent hospital appeals, and of this latest appeal in particular, is valuable evidence in support of the fact that the voluntary hospital is still able to interest the people and to trust to public generosity. The public is still sympathetic towards institutions which show that they deserve support and need it, and no voluntary hospital which is ably managed and has nothing to be ashamed of, need fear that its claims upon voluntary public support will be ignored.

The other lesson is that the personal factor and personal service are still very valuable assets of a hospital. Where an institution can find friends so wholeheartedly energetic in its interests as Prince Francis of Teck, and such zealous workers as his able band of assistants, have proved themselves to be, it need never fear that it will be unable to stand the shocks of those who clamour for the abolition of a system which finds a proof of its success in the fact that our hospitals can still secure such supporters and friends.

